# Quantitative hydro-geophysical analysis of a complex structural karst aquifer in Eastern Saudi Arabia

**DOI:** 10.1038/s41598-019-39192-4

**Published:** 2019-02-26

**Authors:** Mohamed El Alfy, Aref Lashin, Turki Faraj, Abed Alataway, Qassem Tarawneh, Abdelaziz Al-Bassam

**Affiliations:** 1 Prince Sultan Institute for Environmental, Water and Desert Research, King Saud University, Riyadh, 11451 Saudi Arabia; 2 Petroleum and Natural Gas Engineering Department, College of Engineering, King Saud University, Riyadh, 11421 Saudi Arabia; 3 Geology Department, Faculty of Science, Mansoura University, Mansoura, 35516 Egypt; 4 Geology Department, Faculty of Science, Benha University, Benha, 13518 Egypt; 5 National Center for Water Research and Studies (NCWRS), MEWA, Saudi Arabia

## Abstract

The Umm er Radhuma (UER) Formation is a major karst aquifer in Saudi Arabia. This study investigated the hydraulic and petrophysical characteristics of the folded UER carbonate aquifer using integrated hydrological and geophysical logging datasets to understand its complex hydraulic setting as well as detect possible water flow. Petrophysical analysis showed that the UER aquifer has three zones with different lithologic and hydraulic properties. The upper zone attains the best properties with average values of 20%, >100 mD, 3.30 × 10^−5^–1.34 × 10^−3^ m/s, and 1.49 × 10^−3^–6.04 × 10^−2^ m^2^/s, with respect to effective porosity, permeability, hydraulic conductivity and transmissivity. The gamma-ray logs indicate a good fracture system near the upper zone of the UER Formation. Pumping test measurements of transmissivity, hydraulic conductivity and storage coefficients were matched with those from geophysical logs and found to be within the expected range for confined and leaky aquifers. Hydrogeological properties were mapped to detect possible groundwater flow in relation to the dominant structure. The underground water of the folded UER aquifer was forced along meandering flow patterns from W-E to SW-NE through the anticlinal axes. The integrated approach can be further used to enhance local aquifer models and improve strategies for identifying the most productive zones in similar aquifer systems.

## Introduction

Groundwater is considered among the most treasured natural resources, particularly in arid areas. Hydraulic conductivity (K), transmissivity (T), and storage coefficient (S) are key parameters for evaluation of aquifers. Aquifer parameters can be determined using pumping tests, which are costly and only provide local results. Heterogeneity of aquifer sediments, complex geologic settings, and multipart boundaries spatially and temporarily influence aquifer hydraulic parameters^1,2^. However, it is difficult to efficiently characterize these parameters in different aquifer zones. A precise image of the aquifer can be achieved using an integrated approach in which well logging and pumping test data are conjointly interpreted.

Carbonate aquifers have significant secondary porosity because of fractures and solution cavities. They have anisotropic performance because of the consequence of preferential flows through bedding plane fractures and joints, particularly in folded rocks. The magnitude of the aquifer anisotropy depends on the density and spacing of joints which cut across the bedding plain. In these carbonate aquifers, directions of groundwater flow may be difficult to predict, because of their anisotropic nature and existing geologic structures. Groundwater flow may not be perpendicular to lines of equipotential, particularly in karstic areas where conduit systems may act. The directions of maximum and medium K conform to the strike and dip of bedding, respectively, while the minimum K direction is perpendicular to bedding^3^. To estimate aquifer hydraulic parameters, different pumping test methods are used; however, long-duration, step pumping, and recovery tests can be used. Estimation of aquifer hydraulic parameters is affected by aquifer stratigraphic position (unconfined, confined, leaky, and partial penetration vertical anisotropy); therefore, different interpretive techniques are used^4–9^. The estimation of aquifer hydraulic parameters from geophysical measurements has received significant attention over the past two decades^10–12^. An important phase in quantitative hydro-geophysical analysis is the inversion of the aquifer petrophysical properties into the preferred hydraulic parameters^13,14^. Nearly all types of well-logging suites used in hydrocarbon exploration can be implemented in groundwater prospecting^15–24^. The integration of petrophysical analyses and pumping test techniques can be used to enhance understanding of the hydrogeological models through precise detection of a few important aquifer properties, i.e. thickness, porosity, permeability, T, K, etc.^25,26^.

**Figure 1:**
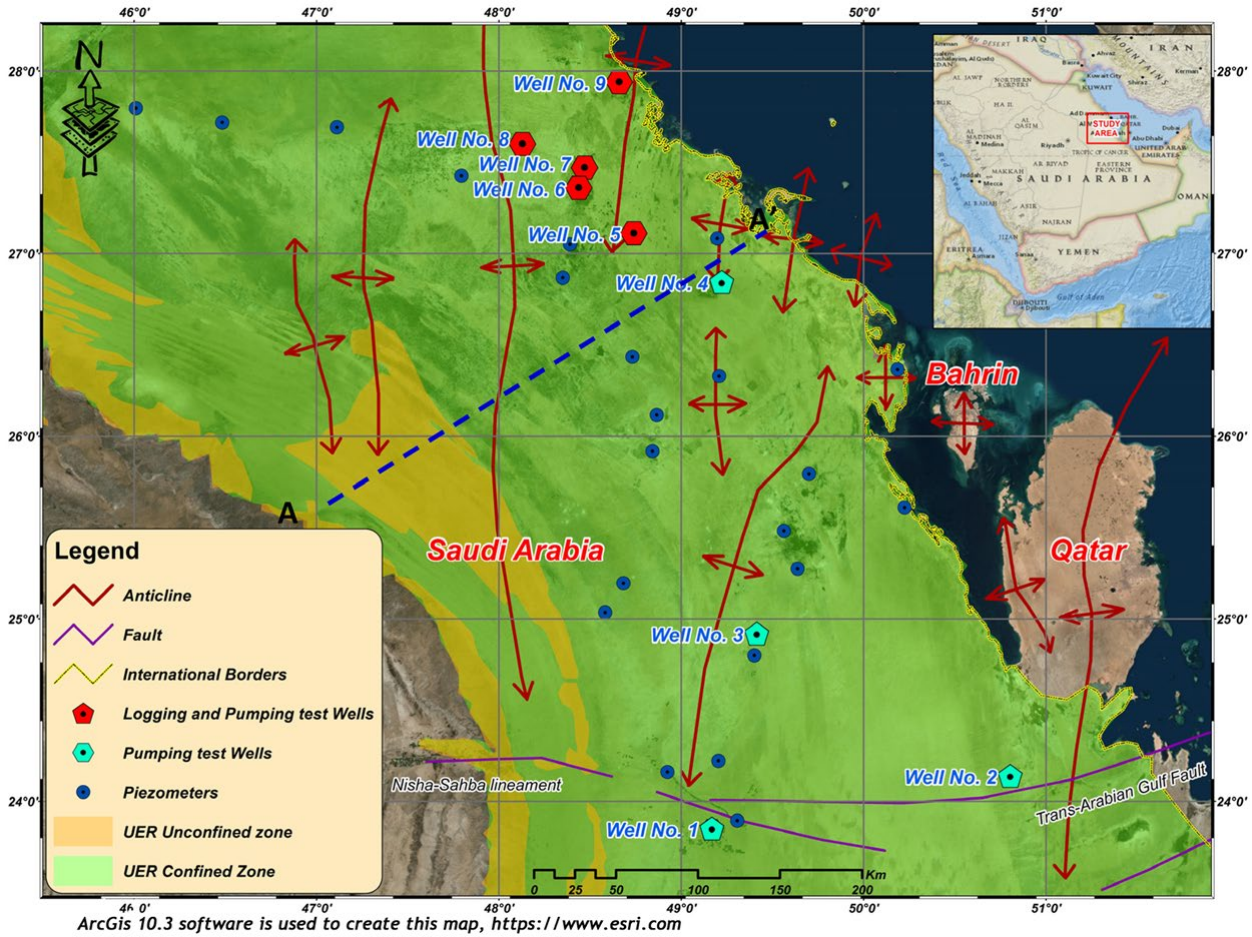
A general map of the study area showing the location of tested wells and the prevailing structural features. ArcGIS 10.3 software is used to create this map, http://www.esri.com.

The study area is located on the peneplain of eastern Saudi Arabia, sloping gently towards the Arabian Gulf (Fig. 1). The main important geomorphic features are wadis, sabkhas, karstic features, and sand dunes. Wadis which are occasionally active during times of intense rains form an important source of groundwater recharge^27^. The joint-flow drainage systems in the study area cause differential dissolution of the carbonate rocks, inducing the development of the eroded karstic terrain. The Umm er Radhuma (UER) Formation is the main water-bearing karst aquifer in the study area. Many anticlinal and synclinal structural features, of great hydraulic importance, are influencing the area. Extensional strain along the anticline axis causes fractional deformation of the bedded rocks which generates water paths even through the impervious layers. Many artesian springs are located along the anticlines forming the natural outflow of the deeper UER aquifer^28^. Natural outflow along the Gulf coast is also recorded where some submarine springs are detected, besides the springs of the island of Bahrain^29^. However, the exact paths and directions of groundwater flow in relation to the properties of the aquifer are still not clearly investigated.

The aim of this study was to use an integrated geophysical well logging and pumping test analysis to characterize the detailed petrophysical properties and understand the complex hydraulic setting of the folded carbonate aquifer of the UER Formation as well as detect possible ground water flow directions.

## Geological and Hydrogeological Settings

**Figure 2:**
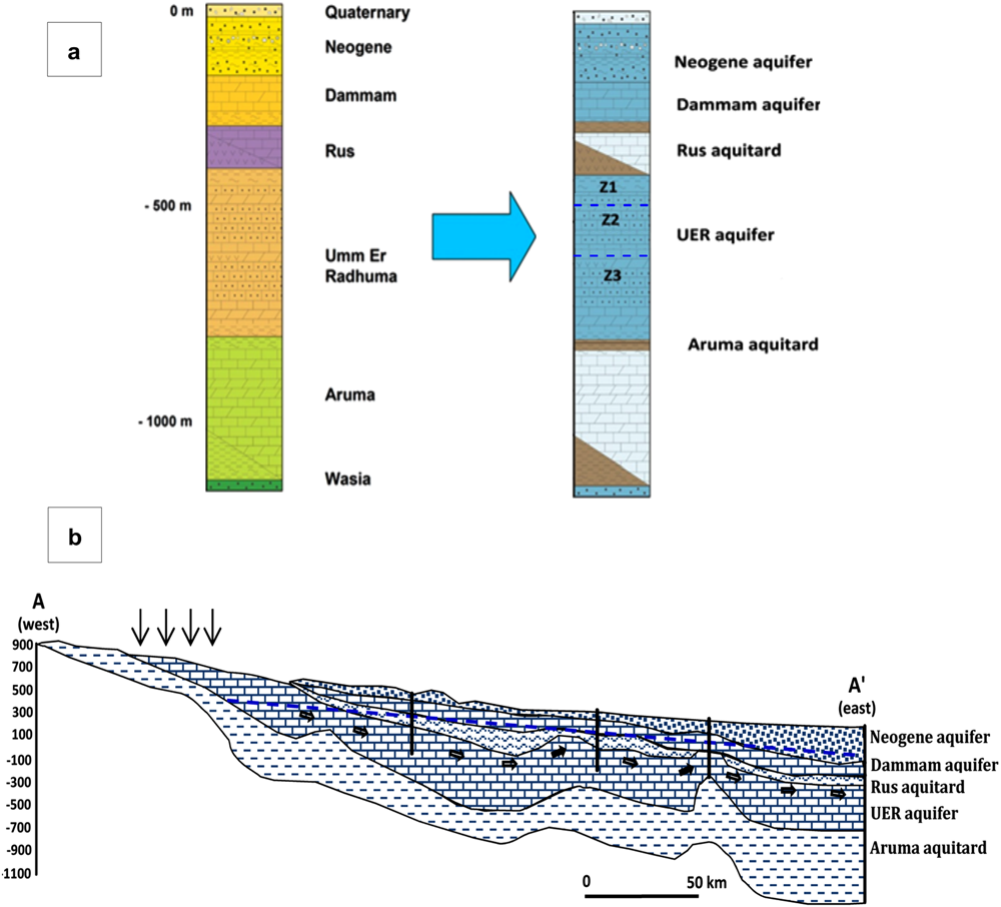
(**a**) A litho-stratigraphic column of eastern Saudi Arabia showing the different rock units (left) and the associated aquifers (right), (**b**) A-A’ generated cross section (See Fig. 1 for location) showing the unconfined and confined zones of UER aquifer as well as groundwater flow directions from west to east. ArcGIS 10.3 software is used to create this cross section, http://www.esri.com.

In eastern Saudi Arabia, the Paleogene sedimentary rock sequence is sub-divided into the UER, Rus, and Dammam formations (Fig. 2a). The UER Formation of the Upper Paleocene–Lower Eocene unconformably overlies the Upper Cretaceous Aruma Formation and underlies the younger Rus and Dammam formations^30,31^. The UER Formation is composed of light-colored foraminiferal aphanitic and calcarenitic limestone, dolomitic limestone, and dolomite that was deposited under marine shelf conditions^32^. Several karstic features are observed in the upper part of these carbonate rocks on the outcrops. Subsurface evidence has also been recorded, in which lost circulation cavities are commonly encountered during drilling^33^. During the Early Eocene, the prevailing arid conditions caused the deposition of the evaporitic Rus Formation. During the Middle Eocene, a marine transgression occurred and the Dammam Formation, composed of limestone and dolomitic limestone, was deposited. Neogene sediments overlie the Dammam Formation consist of friable sandstone, some gravel, and rock fragments. The UER and the overlying Rus, Dammam, and Neogene formations dip slightly eastward, with increasing thickness (up to 800 m), with an average thickness of 400 m. The gently dipping strata are interrupted by a series of asymmetric anticlines and synclines with axes plunging to the N and NE; these folds are bounded by high-angle reverse faults (Fig. 1). Figure 2b shows the influence of aquifer dipping and anticlinal structures on groundwater flow. The hydrogeological cross section A-A’ was constructed using the geologic map, lithologic logs, and hydrogeologic data obtained from well logs. This cross section shows the unconfined and confined zones of UER aquifer as well as groundwater flow directions from west to east. The hydraulic connection between UER and the Dammam and Neogene aquifers is identified in areas where the Rus aquitard is absent above the anticlinal crests (Fig. 2b).

The UER aquifer lies atop of the underlying Aruma aquitard. The dissolution of the UER carbonates has developed various karstic features; consequently, porosity and permeability are increased, and groundwater flows quickly downward, dipping eastward. In the western part at the outcrop areas of the UER aquifer, an unconfined condition prevails. Far to the east near the Arabian Gulf, the UER aquifer exhibits a confined condition. Near the Arabian Gulf, these rocks are found at great depths and are of a lower porosity and permeability; therefore groundwater flows slowly eastward. The Rus Formation comprises anhydrite, limestone, and shale, and forms an aquitard overlying the UER with many discontinuities related to erosion over the anticlinal structures. Because of the dissolution of the Rus evaporites, some zonal areas may act as hydraulic channels connecting the UER and the overlaying aquifers. The differences in the hydraulic heads between the UER and Dammam and Neogene aquifers cause a significant exchange of groundwater. The upward leakage of UER water forms the coastal sabkhas near the Arabian Gulf, and the Al-Ahassa springs^34^.

Because of the present arid climate, minor modern recharge is recorded, restricted to UER outcrops, particularly when UER is in direct contact with sand dunes. The main groundwater recharge of the UER aquifer occurred during the Pleistocene. Stable isotopes show that the groundwater age of the UER aquifer in the outcrop area is recent, while its age in its eastern part is 20,000–26,000 years^35–37^. Negative δ^18^O values < −5.5% have been reported from the Ghawar anticlinal structure groundwater, where upward leakage of deeper groundwater from the UER aquifer is assumed. The hydraulic parameters of the UER aquifer were identified in different locations by GTZ (2006)^38^. They are relatively moderate to low, with average values of 1.6 × 10^−5^ m/s and 2.0 × 10^−3^ m^2^/s for K and T, respectively^38^. Generally, groundwater quality of the UER aquifer deteriorates eastward towards the Arabian Gulf from <1000 mg/1 near the outcrop to 6000 mg/1 along the coastal area^38,39^. Relatively better water quality is observed over the domal structural highs because of the absence of the gypsum and anhydrite of the Rus Formation.

## Results

### Geophysical Logging Analysis

The well-logging analysis clarified the petrophysical and hydrogeological characteristics of the UER aquifer. A number of cross plots, histograms, and vertical analogs were constructed and interpreted. Standard Archie equation was applied for fluid content determination and differentiation between producible and connate water in clean zones of the carbonate aquifer. Meanwhile, a modified form of equation was applied to compensate for the shale content, which acts as another electrically conductive material beside the formation water^40^.

#### Aquifer zonation

**Figure 3:**
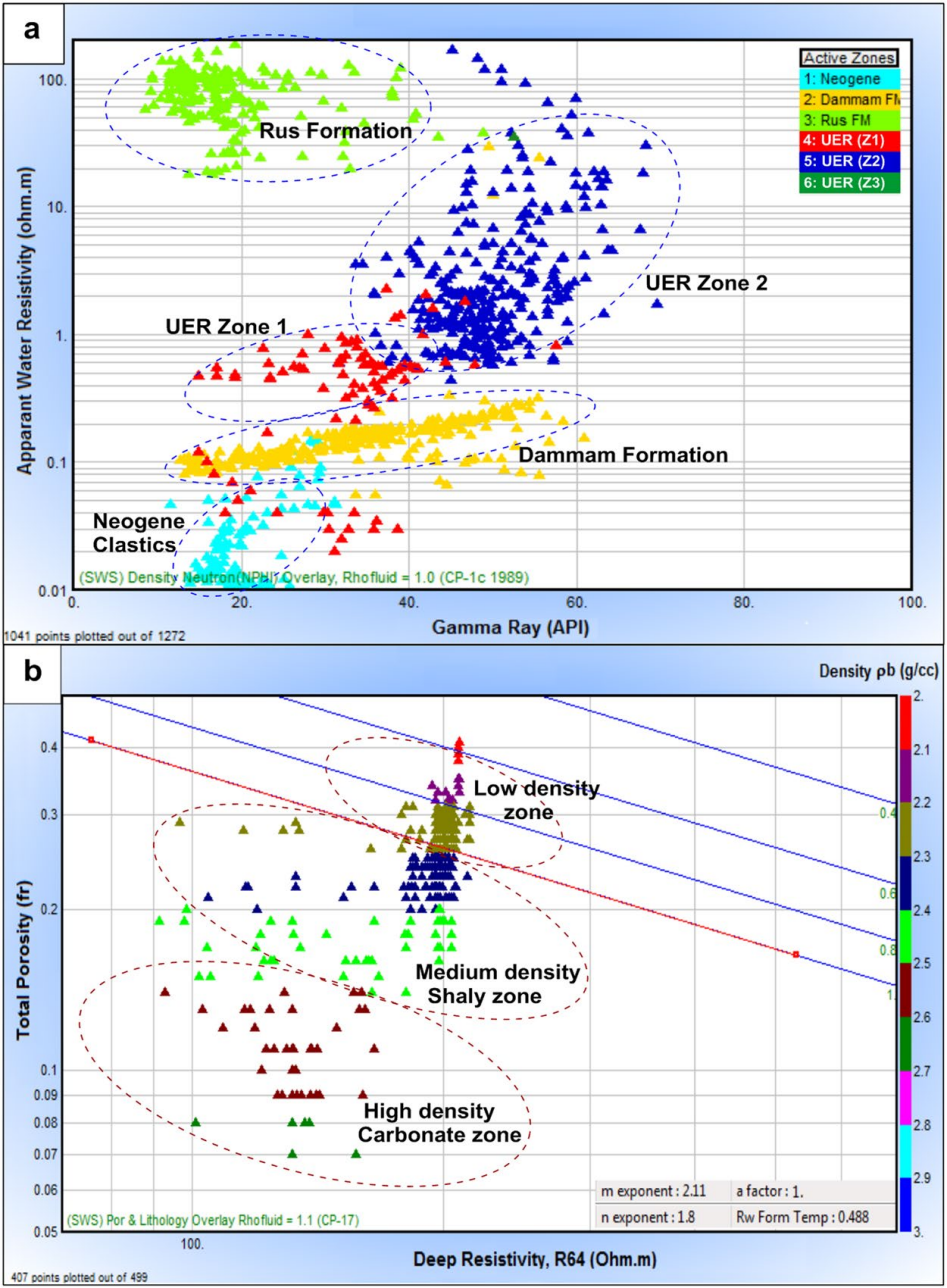
(**a**) Gamma ray–apparent water resistivity plot of all rock units, and. (**b**) Resistivity–porosity Pickett plot of the UER aquifer, well No. 6.

Based on the deduced petrophysical parameters, a detailed zonation of the UER aquifer was established. It was divided into three main zones, two upper and lower zones (Z1 and Z3) of considerable thickness and a thick middle zone (Z2). Figure 3a shows the apparent water resistivity (Rwa)-gamma ray (GR) cross plot of the different rock units encountered in well No. 6. Although these rock units are characterized by low GR, a wide range of Rwa (0.01–200 Ohm.m) was observed, leading to good differentiation between the rock units. Low Rwa values (<1.0 Ohm.m) were assigned to the Neogene clastics, Dammam Formation and UER Formation (Z1); these rock units are considered clean to shaly and nearly share the same range of GR (15–55 API). On the other hand, the Rus Formation exhibits a higher range of Rwa (20–200 Ohm.m) and a lower GR response because of its evaporitic nature. Meanwhile, the low hydraulic properties of the UER Formation (Z2) are reflected in its high GR and the intermediate-to-high apparent water resistivity (Rwa, 0.8–100 Ohm.m).

The Pickett plot shown in Fig. 3b ensures the water-bearing nature of the UER Formation and assigns m and n parameters of 2.11 and 1.8, respectively. Most of the data points were clustered below the 100% water saturation (WS) line, constituting two zones of medium-density shaly and high-density carbonate lithology. Few low-density points were clustered between the WS lines of 100% and 60%, indicating a shaly zone enriched by organic materials (high resistivity and low density). This supports the zonation of the UER aquifer into three different zones based on its lithological content and fluid characteristics.

#### Lithology and shale volume

**Figure 4:**
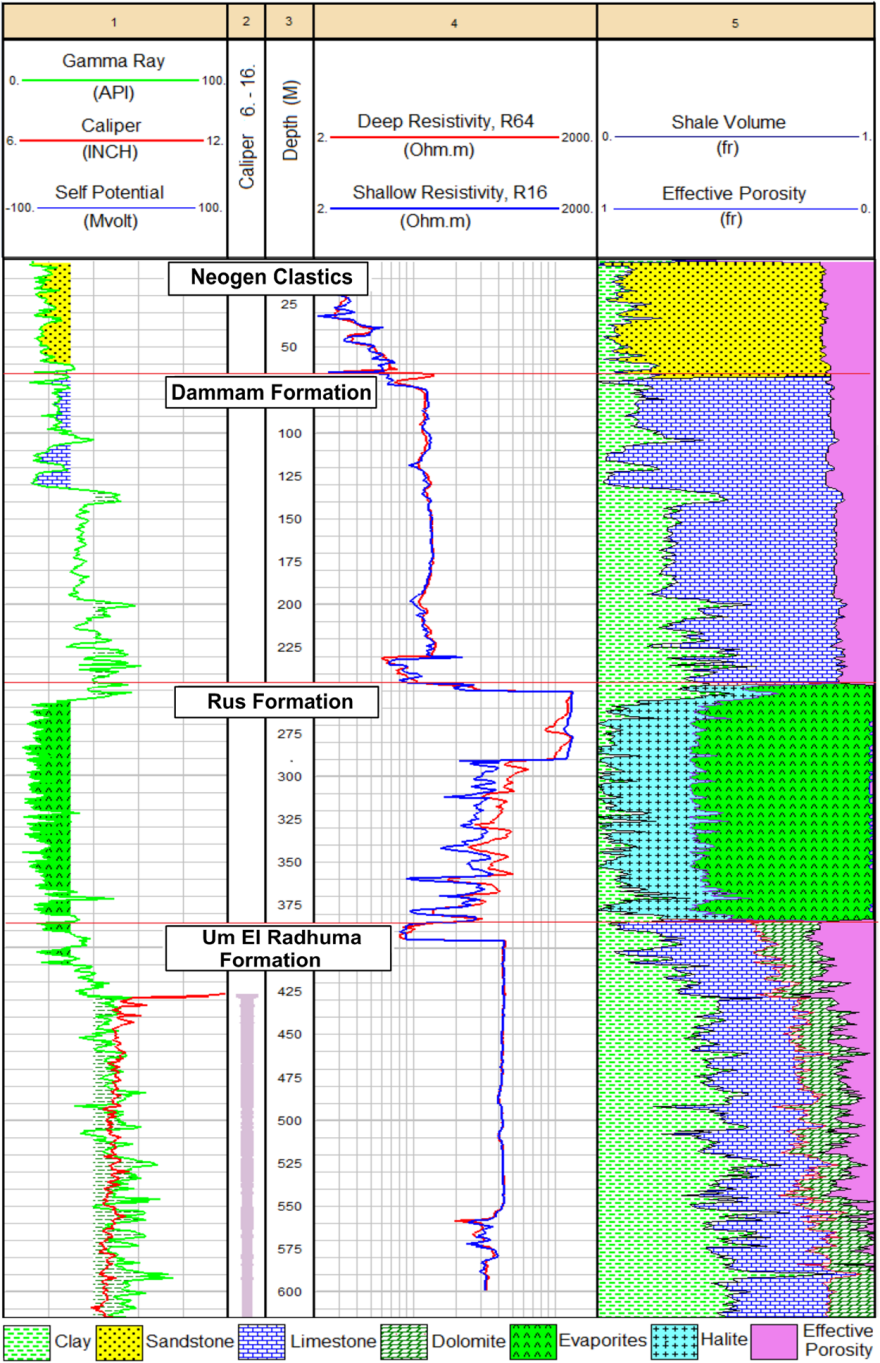
Composite log plot of well No. 6 showing the generated lithology (Track 5) and the preliminary logs used in the interpretation (Tracks 1, 2 and 4).

**Figure 5:**
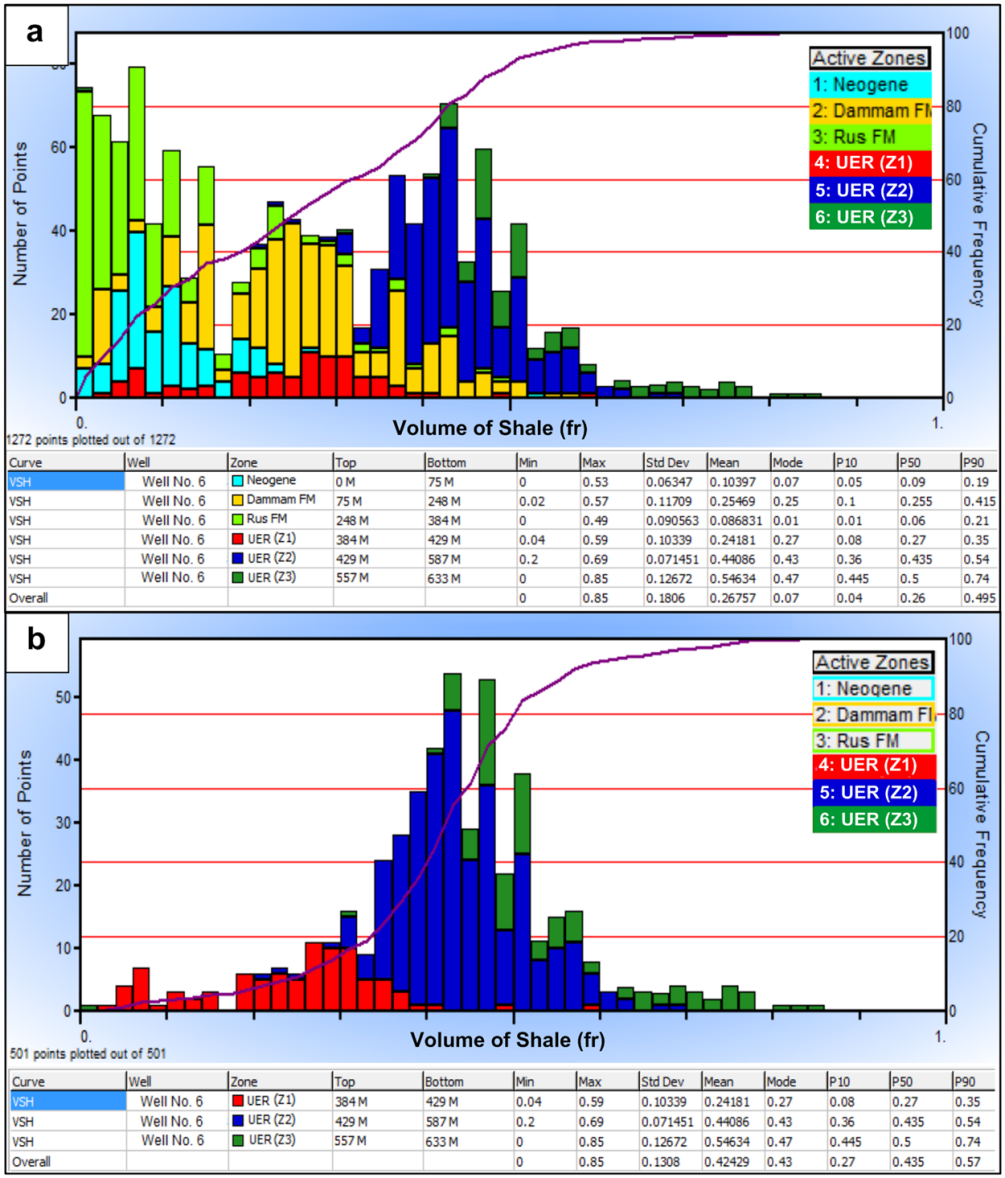
Shale volume histograms for well No. 6; (**a**) all rock units and (**b**) UER aquifer. The clean shale nature of the Neogene and Rus formations is clearly seen (**a**). The three zones of the UER Formation show widespread shale distribution; however, zone 1 (Z1) has the lowest shale volume (**b**).

Detailed petrophysical analysis was performed to detect the main lithological composition of the different rock units generating lithological columns. These columns were verified using the description of the rock cuttings and drilling samples. Track 5 of well No. 6 was selected as an example to demonstrate the log-derived lithology (Fig. 4). It shows that limestone carbonates constitute the main lithological component of the UER Formation (70–75%) followed by dolomite (25–30%), with a relative abundance in the Z1 zone. Quantitatively, both limestone and dolomite share 50–60% of the volumetric analysis of the aquifer, while the remaining (40–50%) is represented by the shale volume and pore spaces. The Rus Formation, which is mainly evaporitic in nature, is represented by halite and anhydrite with 1:4 proportional ratios. The Dammam Formation is dominated by a limestone lithology associated with low shale content and low resistivity response as compared to the UER Formation. The Neogene rocks are represented by clean clastics, mainly sandstone in composition (Fig. 4). The constructed shale volume histogram of well No. 6 in Fig. 5a illustrates the clean-bearing characteristics and low shale content of the Neogene units and the Rus and Dammam formations. On the other hand, Fig. 5b shows the shale content histogram of the three identified zones of the UER Formation. It shows that Z1 has the lowest shale content followed by Z2 and Z3 in an increasing order with depth. The average values of shale volume for the three zones are 24%, 44%, and 55%, respectively.

#### Porosity and facture system identification

**Figure 6:**
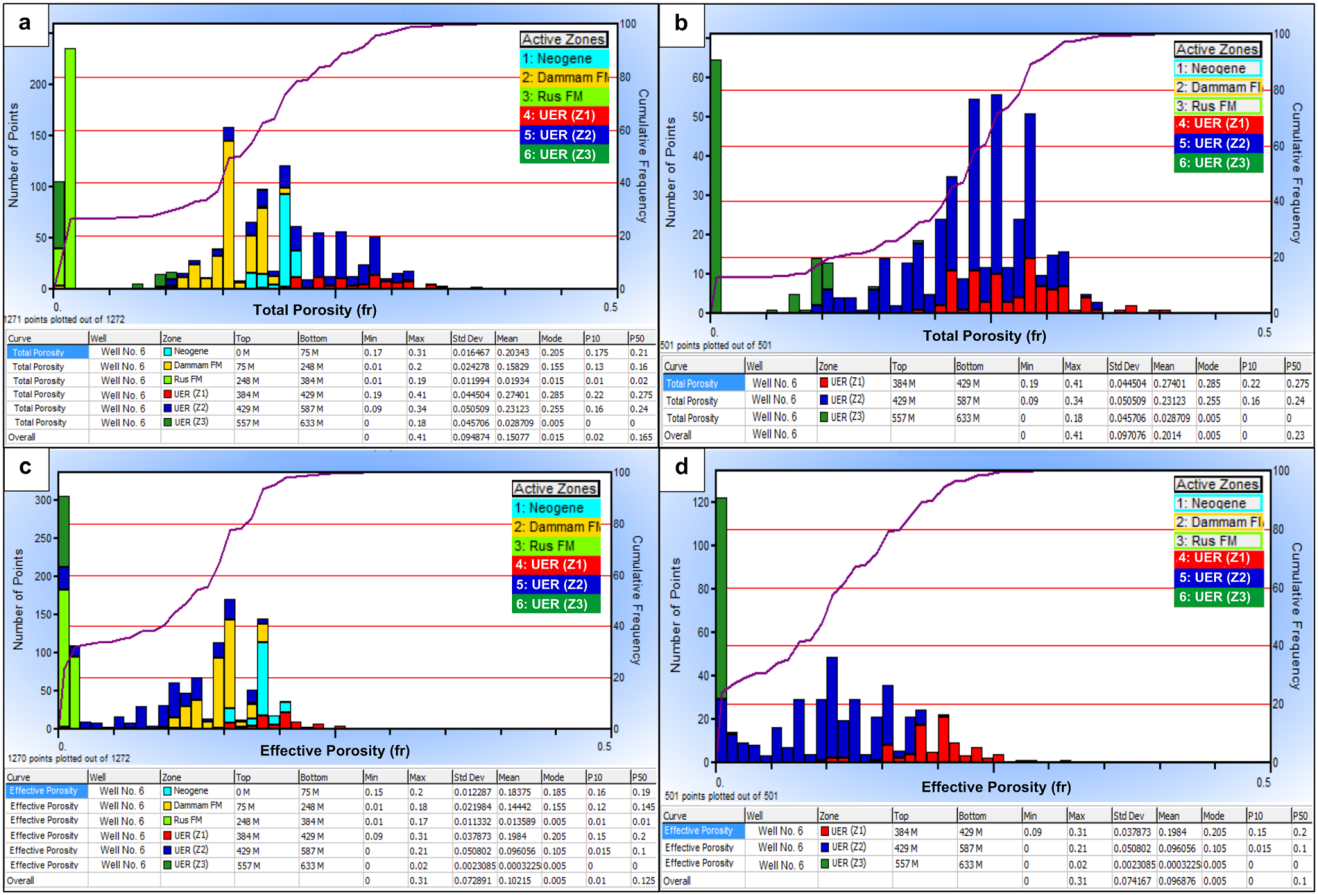
Total and effective porosity histograms for well No. 6; (**a**) Total porosity histogram for all rock units, (**b**) Total porosity histogram of UER aquifer, (**c**) Effective porosity histogram of all rock units and, (**d)** Effective porosity histogram of UER aquifer. Z1 of UER shows good total and effective porosity ranges as compared to those of the other zones.

A wide range of total porosity distributions is observed for the different rock units encountered in the study area (Fig. 6a). The Rus Formation evaporites have very low porosity values of 1%, and the Dammam carbonates and Neogene sediments exhibit moderate porosity distribution (12–22%). The highest record of total porosity was found in the UER Z1 and Z2 zones with average values of 27% and 23%, respectively (Fig. 6b).

**Figure 7:**
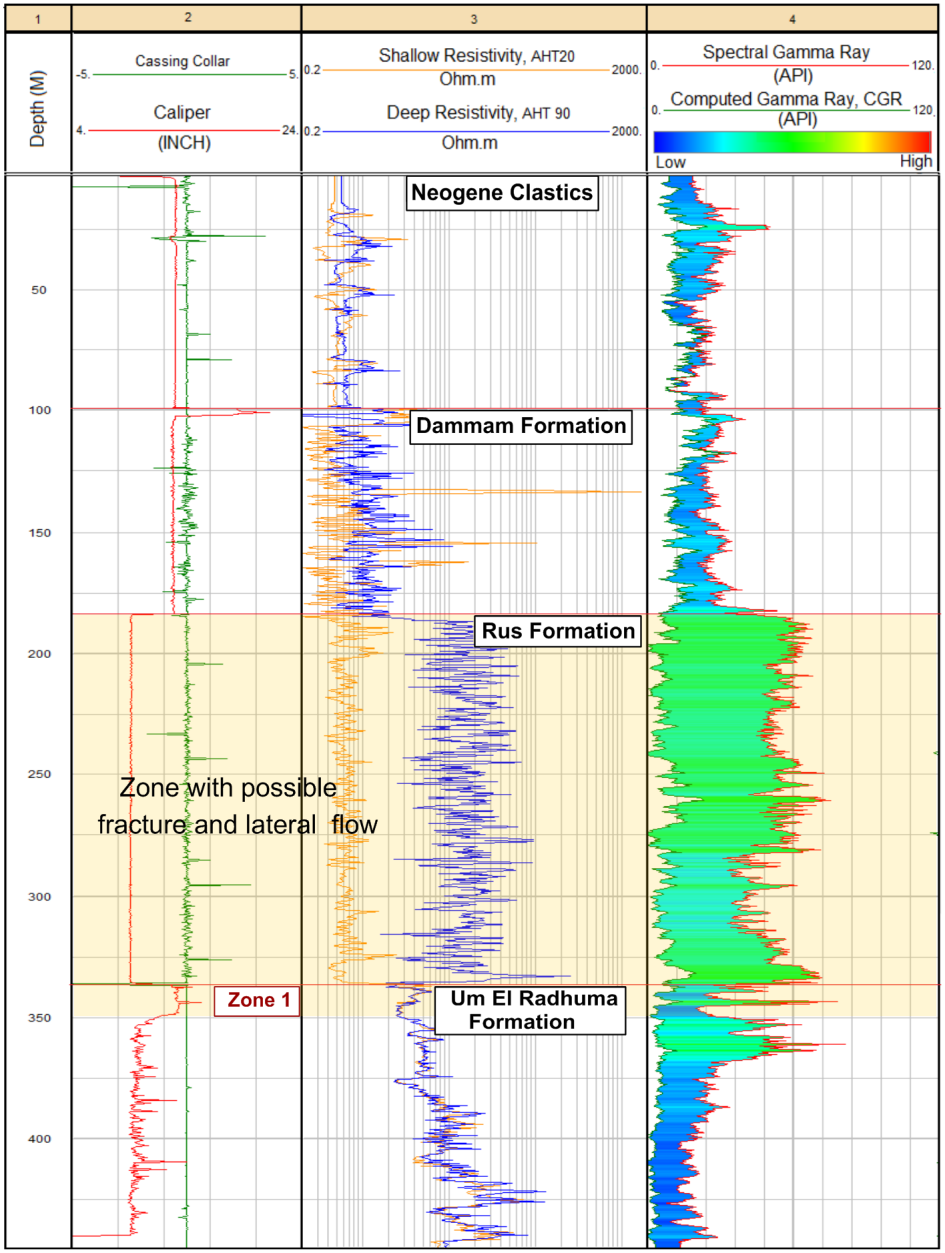
SGR and CGR–resistivity curve overlays of well No. 5 showing the possible fracture system in front of the Rus Formation and the topmost of the underlying zone (Z1) of the UER aquifer.

The effective porosity distribution (*ϕ*_*eff*_) is illustrated in Fig. 6(c,d). Both of the Z1 and the Neogene clastics show good effective porosity; however, the average value of Z1 (20%) is higher than that of the Neogene rocks (18%). The Dammam Formation and UER Formation Z2 exhibit average lower values of 14% and 10%, respectively. Meanwhile, very low effective porosity was found for Z3 of the UER Formation and the Rus Formation (*ϕ*_*eff*_ < 2%). Figure 7 shows the spectral gamma ray (SGR) and computed gamma ray (CGR) vs. resistivity curve overlays of well No. 5. A wide separation is notable in Track 4 between the SGR and CGR curves in front of the Rus Formation and uppermost part of the UER Formation. This was supported by the behavior of the resistivity logs (separation between shallow and deep curves, Track 3) and caliber logs (Track 2). This is a good indication of the presence of a strong fracture system with possible lateral flow.

#### Petrophysical Analogs

**Figure 8:**
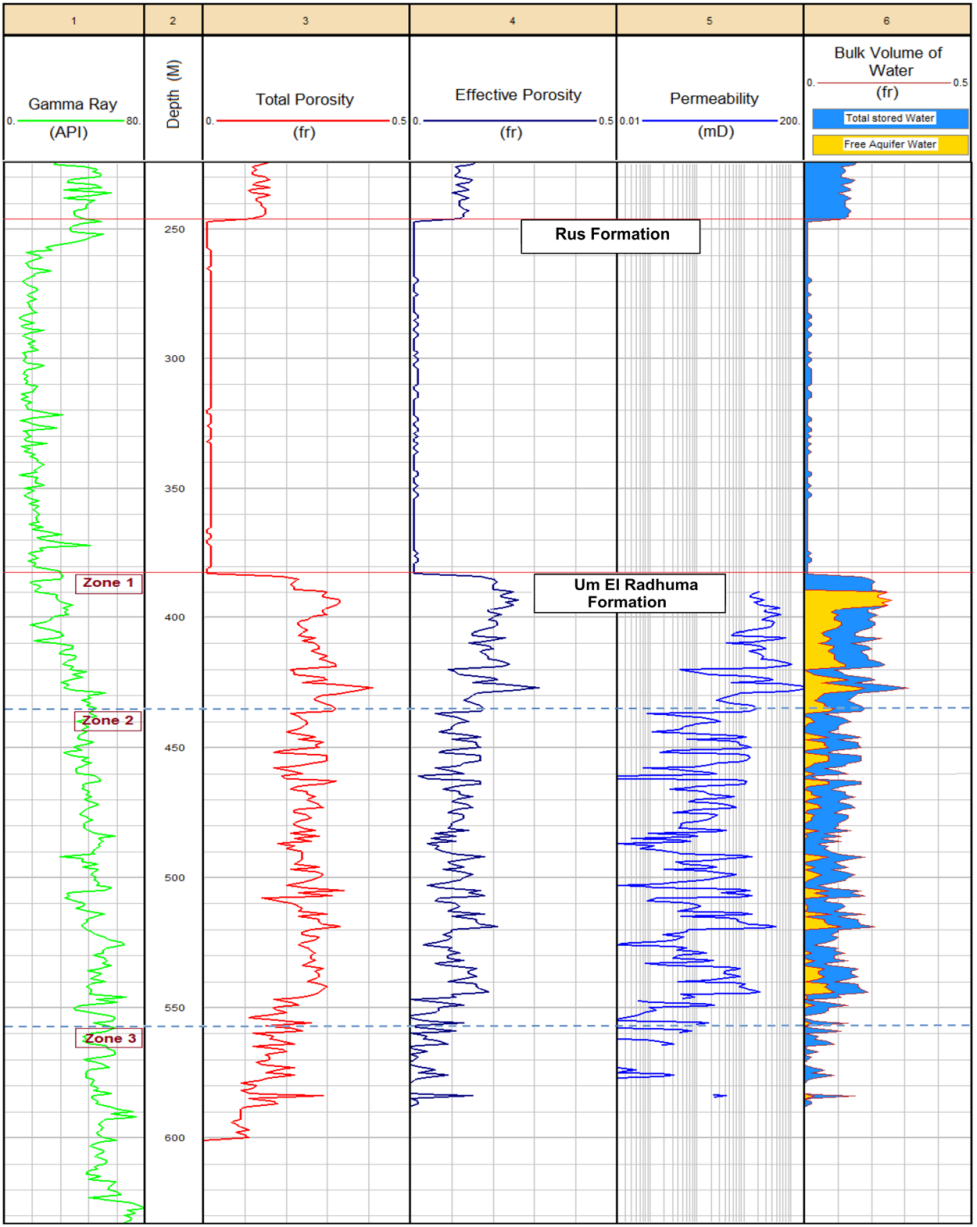
The log-derived total porosity, effective porosity, permeability and bulk water volume of well No. 6. No pore system or fluid content are indicated in front of the Rus Formation. The upper zone of UER aquifer (Z1) is the best hydraulic zone with good porosities (total and effective porosity), high specific yield and high permeability.

Petrophysical analysis of the UER Formation and the overlying evaporitic Rus Formation is shown in Fig. 8. It shows the log-derived total porosity, effective porosity, permeability and bulk volume of water. Another complied plot integrating the above-mentioned petrophysical parameters with the aquifer resistivity (deep & shallow), apparent water resistivity, shale volume and lithology is represented at the Supplementary Information (Fig. S1). The lithological components of the Rus Formation are represented mainly by anhydrite and halite with very low shale content. The clean characteristics and the evaporitic composition of the Rus Formation are reflected in the low GR response and high deep and shallow resistivity logs (Track 1, Fig. 8 and Track 3, Fig. S1, Supplementary Information). Negligible pore spaces with no permeability and bulk volume of water are recorded in front of the Rus Formation (Tracks 3 to 6, Fig. 8). Considering the UER Formation, it seems clear that the upper zone (Z1) is the best hydraulic zone. It has the highest total porosity (*ϕN*_*Tot*_ > 0.28 fr), effective porosity (*ϕN*_*eff*_ > 0.18 fr) and bulk volume of stored water (high specific yield) associated with a high record of permeability (*k* > 100 mD). Less separation is indicated between the total and effective porosity curves (Track 4, Fig. S1) indicating a lesser effect of the shale and that most of the pores are connected. The free aquifer water is much higher in this zone as compared to that of the underlying zones; hence, there is good water production. The middle zone (Z2) is a thicker zone (approximately 128 m in thickness) with lower hydraulic properties (high specific retention). Lower values of porosities (*ϕN*_*Tot*_ 0.20–0.28 & *ϕN*_*eff*_ 0.05–0.15 fr) and permeability (*k* < 10 mD) associated with moderate content of total stored and free water production were found. The third zone (Z3) has low hydraulic parameters; it has less effective pore spaces, less permeability and very limited free water content (high specific retention).

#### Log-derived hydrogeological parameters

**Figure 9:**
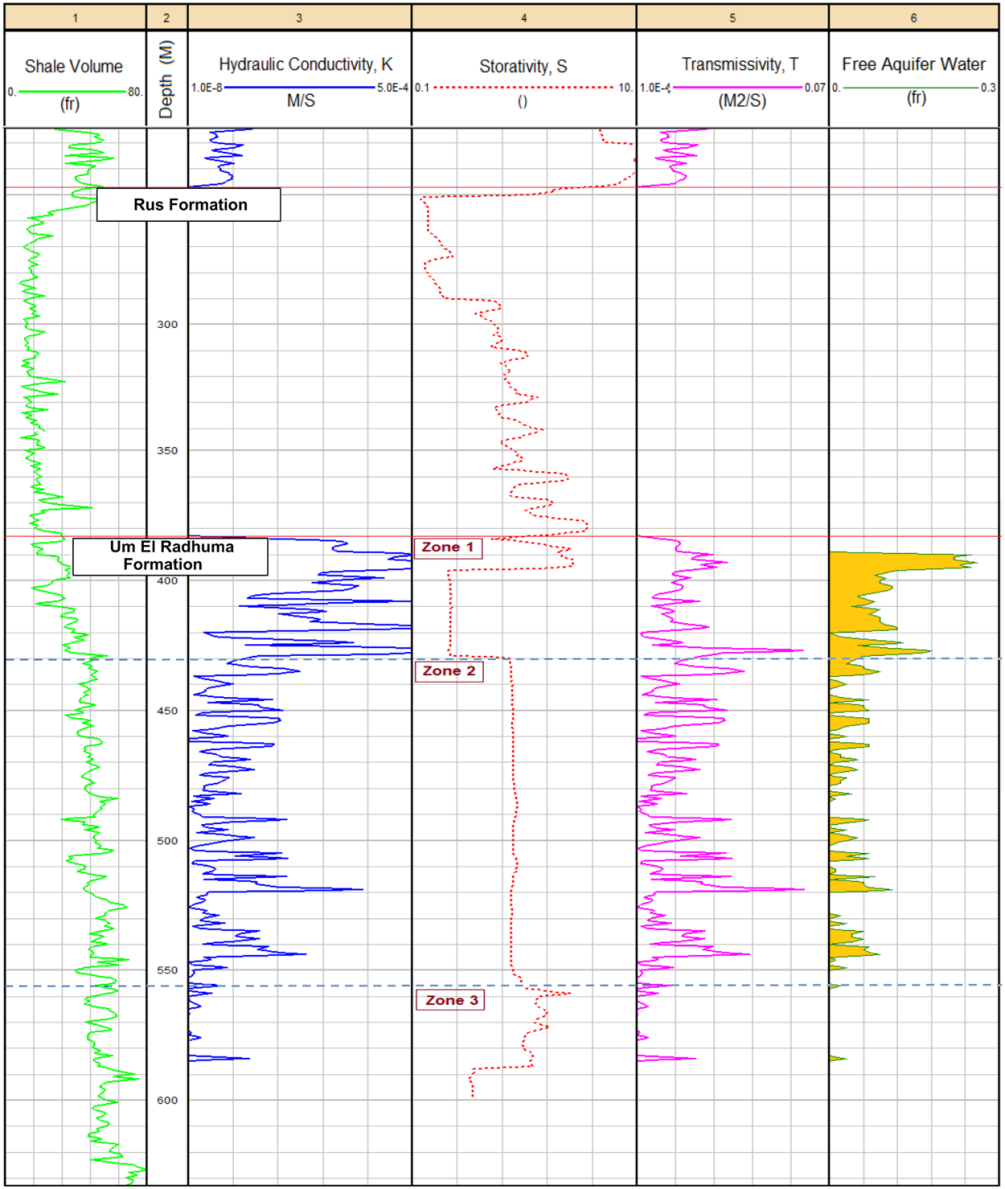
The hydraulic properties of the UER aquifer as inferred from logging analyses of well No. 6. Tracks 3, 4 and 5 represent the log-derived K, S, and T, respectively (see Table 3 for associated values). The aquifer bulk volume of water is indicated in Track 6.

**Figure 10:**
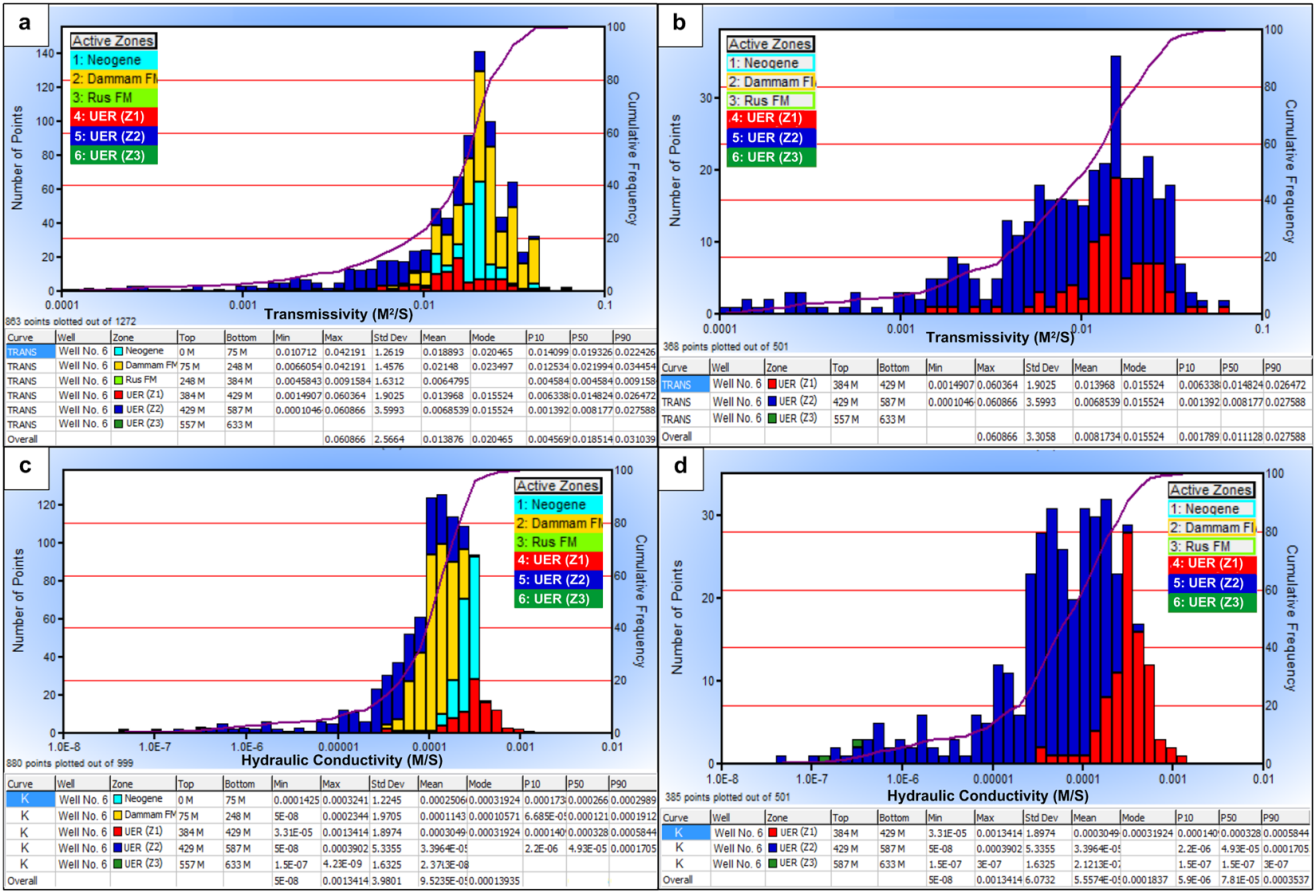
(**a**) Log-derived T histogram of all rock units, (**b**) log-derived T histogram of the UER aquifer, (**c**) log-derived K histogram of all rock units, and (**d**) log-derived K histogram of the UER aquifer of well No. 6. ArcGIS 10.3 software is used to create this map, http://www.esri.com.

Figures 9 and 10 show the vertical analog of the log-derived hydraulic properties of the UER Formation and the overlaying Rus Formation, as well as the zonal histograms for the T and K. Table 1 summarizes the log-derived hydraulic parameters of the three zones of the UER Formation. Furthermore, Figure S2 (Supplementary Information) represents the log-derived hydraulic parameters as correlated with other driven petrophysical parameters (lithology, porosities, resistivity, and shale volume) for better integrating the whole characteristics of the aquifer. It can be inferred from Fig. 9 and Table 1 that the first zone of the UER Formation (Z1) exhibits the best aquifer properties with the highest K, T, and S ranges of 3.30 × 10^−5^−1.34 × 10^−3^ m/s, 1.49 × 10^−3^–6.04 × 10^−2^ m^2^/s, and 5.02 × 10^−4^–4.57 × 10^−4^, respectively. The specific yield is high, where the bulk volume of free producible water reaches up to 0.22 fr in the upper part of this zone (Fig. 9, Track 6). The derived K is comparable to the karstic and reef limestone lithology of Domenico and Schwartz 1990 (Table 2). The lithological content shows much more limestone on the expanse of dolomite with little shale (Track 8, Fig. S2). The second zone (Z2) shows ranges of 5.01 × 10^−8^–3.9 × 10^−6^ m/s, 1.05 ×  10^−4^–6.09 × 10^−2^ m^2^/s, and 1.38 × 10^−3^–1.25 × 10^−3^ for the K, T, and S, respectively. This zone has intermediate petrophysical and hydraulic properties with small specific yield and limited free aquifer water (>0.05 fr). The derived K is similar to that provided by Domenico and Schwartz (1990) for the limestone and dolomite lithology in Table 2. The general ranges of K (5.01 × 10^−8^–1.34 × 10^−3^ m/s), T (1.05 × 10^−4^–6.04 × 10^−2^ m^2^/s), and S (5.02 × 10^−4^–1.25 × 10^−3^) for these two zones (Z1 and Z2) are similar to those of GTZ (2006). The third zone (Z3) has low hydraulic properties and thus limited water production can originate through this zone. The effective porosity and permeability are greatly reduced because of the increased shale content which increases aquifer specific retention (Tracks 4,5 Fig. 8 and Track 1 Fig. 9).

**Table 1:** Ranges of log-derived hydraulic properties of the UER aquifer.

Zone	Hydraulic Conductivity (m/sec)	Transmissivity (m^2^/sec)	Storativity	Specific Storage
Zone 1	3.30 × 10^−5^–1.34×10^−3^	1.49 × 10^−3^–6.04 × 10^−2^	5.02 × 10^−4^–4.57 × 10^−4^	1.02 × 10^−5^–1.12 × 10^−5^
Zone 2	5.01 × 10^−8^–3.9 × 10^−4^	1.05 × 10^−4^–6.09 × 10^−2^	1.38 × 10^−3^–1.25 × 10^−3^	9.8 × 10^−6^–1.08 × 10^−5^
Zone 3	4.2 × 10^−9^–1.50 × 10^−7^	1.80 × 10^−7^–6.45 × 10^−6^	4.24 × 10^−4^–4.21 × 10^−4^	9.80 × 10^−6^–9.87 × 10^−6^

**Table 2:** Most common representative values of K for carbonate aquifers and other sedimentary rocks^6^.

Rock type	Hydraulic Conductivity (m/sec)
Karst and reef limestone	1 × 10^−6^ to 2 × 10^−2^
Limestone, dolomite	1 × 10^−9^ to 6 × 10^−6^
Sandstone	3 × 10^−10^ to 6 × 10^−6^
Siltstone	1 × 10^−11^ to 1.4 × 10^−8^
Salt	1 × 10^−12^ to 1 × 10^−10^
Anhydrite	4 × 10^−13^ to 2 × 10^−8^
Shale	1 × 10^−13^ to 2 × 10^−8^

### Estimating hydraulic parameters from pumping tests

**Figure 11:**
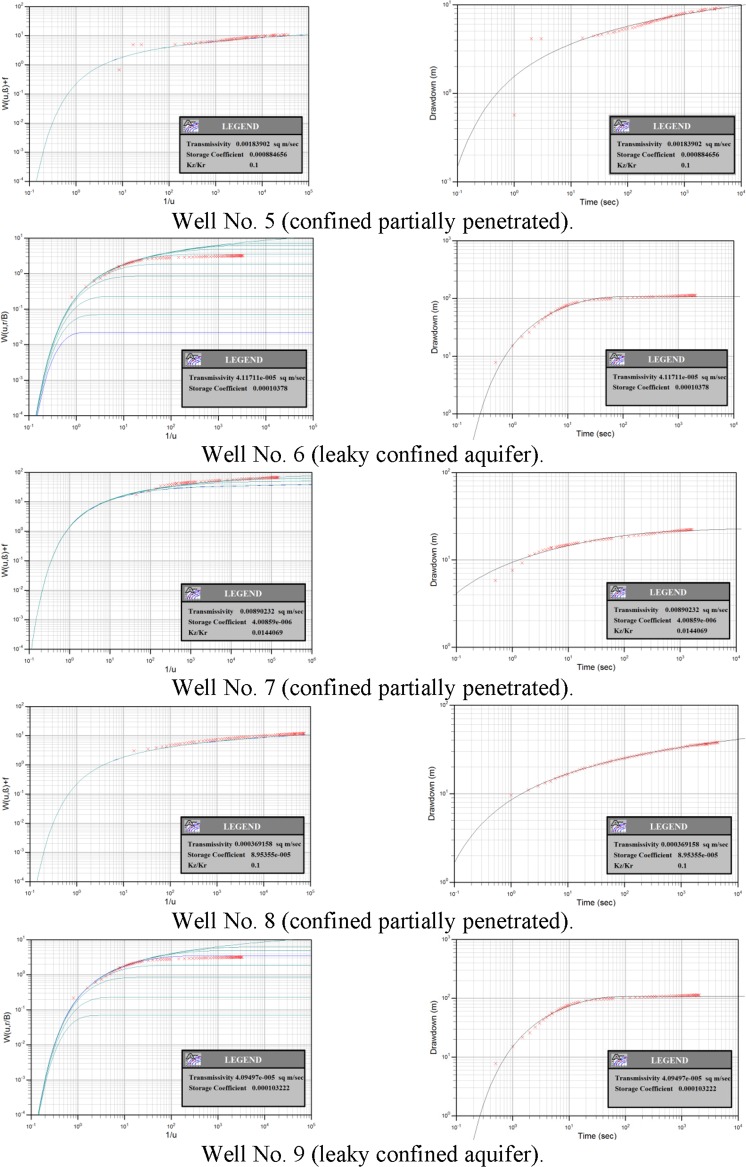
Time-drawdown fitted models using long-duration pumping test data of four wells tapping the UER aquifer. The aquifer nature is confined partially penetrated to leaky confined. ArcGIS 10.3 software is used to create this map, http://www.esri.com.

**Figure 12:**
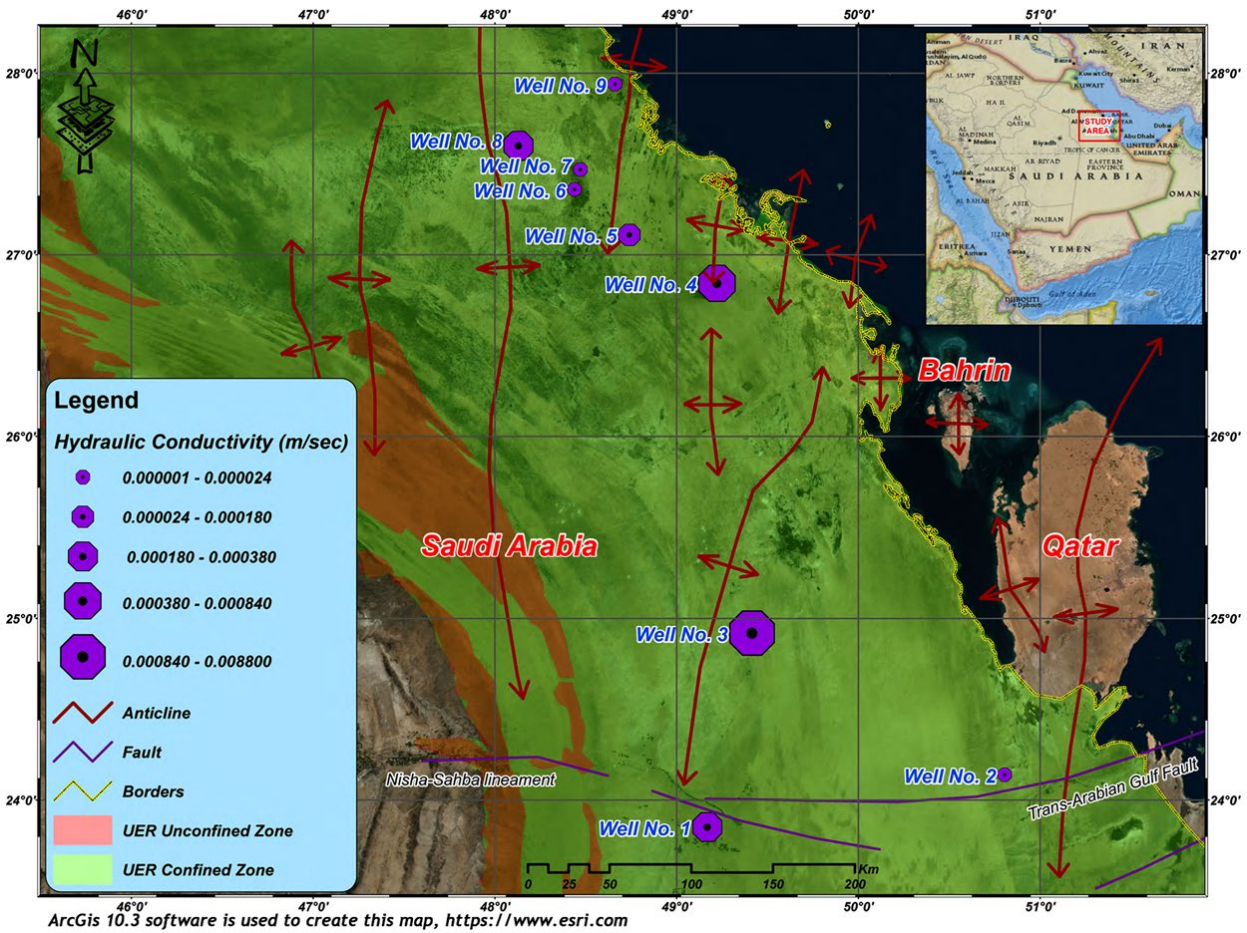
K values of the UER aquifer in the study area. The wide range of K is often above or near the anticlinal structures (see wells No. 3, 4 and 8) and can be attributed to permeable zones formed by the karstification process associated with a high density of fractures and joints. ArcGIS 10.3 software is used to create this map, http://www.esri.com.

According to the hydrogeological setting, the UER aquifer is defined as a leaky unconfined to confined aquifer. Unconfined conditions occur at the outcrop areas to the west, and for some distance to the east, until the piezometric surface intersects with the confining Rus, Dammam, and Neogene beds. In the central and eastern areas, this aquifer is found under confined conditions, and artesian confined conditions occur near the Arabian Gulf. K, T, and S of the UER aquifer were calculated using the long-duration pumping test data from nine wells. The fitted models of the tested nine wells show that the tested K values vary between 6.3 × 10^−7^ and 8.8 × 10^−3^ m/s with an average of 1.1 × 10^−3^ m/s (Fig. 11 and Table 3). The wide range in the K values can be interpreted to be a result of permeable zones due to the karstification process associated with the rapid changes in the high density of fractures and joints, particularly above or near the anticlinal structures as recorded in wells No. 3, 4, and 8 (Fig. 12). In general, the average calculated hydraulic parameter using the long-duration pumping tests correlated with those of the well-logging analyses.

**Table 3:** Summary of the calculated hydraulic parameters of the UER aquifer.

Well ID	Transmissivity (m^2^/sec)	Hydraulic Conductivity (m/sec)	Storage Coefficient
Well No. 1	2.1 × 10^−2^	3.8 × 10^−4^	2.1 × 10^−6^
Well No. 2	4.7 × 10^−4^	4.7 × 10^−6^	2.0 × 10^−4^
Well No. 3	2.8 × 10^−1^	8.8 × 10^−3^	1.6 × 10^−4^
Well No. 4	7.4 × 10^−2^	1.1 × 10^−3^	2.9 × 10^−4^
Well No. 5	1.8 × 10^−4^	9.8 × 10^−6^	8.8 × 10^−4^
Well No. 6	4.1 × 10^−5^	6.3 × 10^−8^	1.0 × 10^−4^
Well No. 7	8.9 × 10^−4^	1.4 × 10^−6^	3.0 × 10^−6^
Well No. 8	3.7 × 10^−4^	1.6 × 10^−6^	9.0 × 10^−5^
Well No. 9	4.1 × 10^−5^	6.3 × 10^−7^	1.0 × 10^−4^
Minimum	4.1 × 10^−5^	6.3 × 10^−7^	2.1 × 10^−6^
Maximum	2.8 × 10^−1^	8.8 × 10^−3^	8.8 × 10^−4^
Average	4.2 × 10^−2^	1.1 × 10^−3^	2.1 × 10^−4^
Standard Deviation	9.3 × 10^−2^	2.9 × 10^−3^	2.7 × 10^−4^

**Figure 13:**
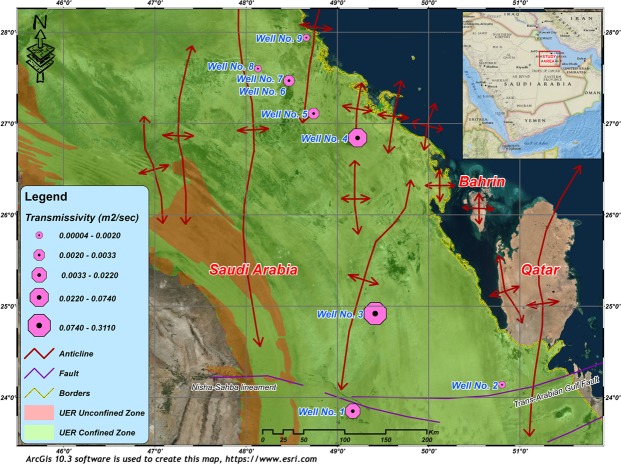
T values of the UER aquifer in the study area. The increasing trend of the T follows the same directions as K (Fig. 12). Lower values were recorded in the synclinal troughs. ArcGIS 10.3 software is used to create this map, http://www.esri.com.

**Figure 14:**
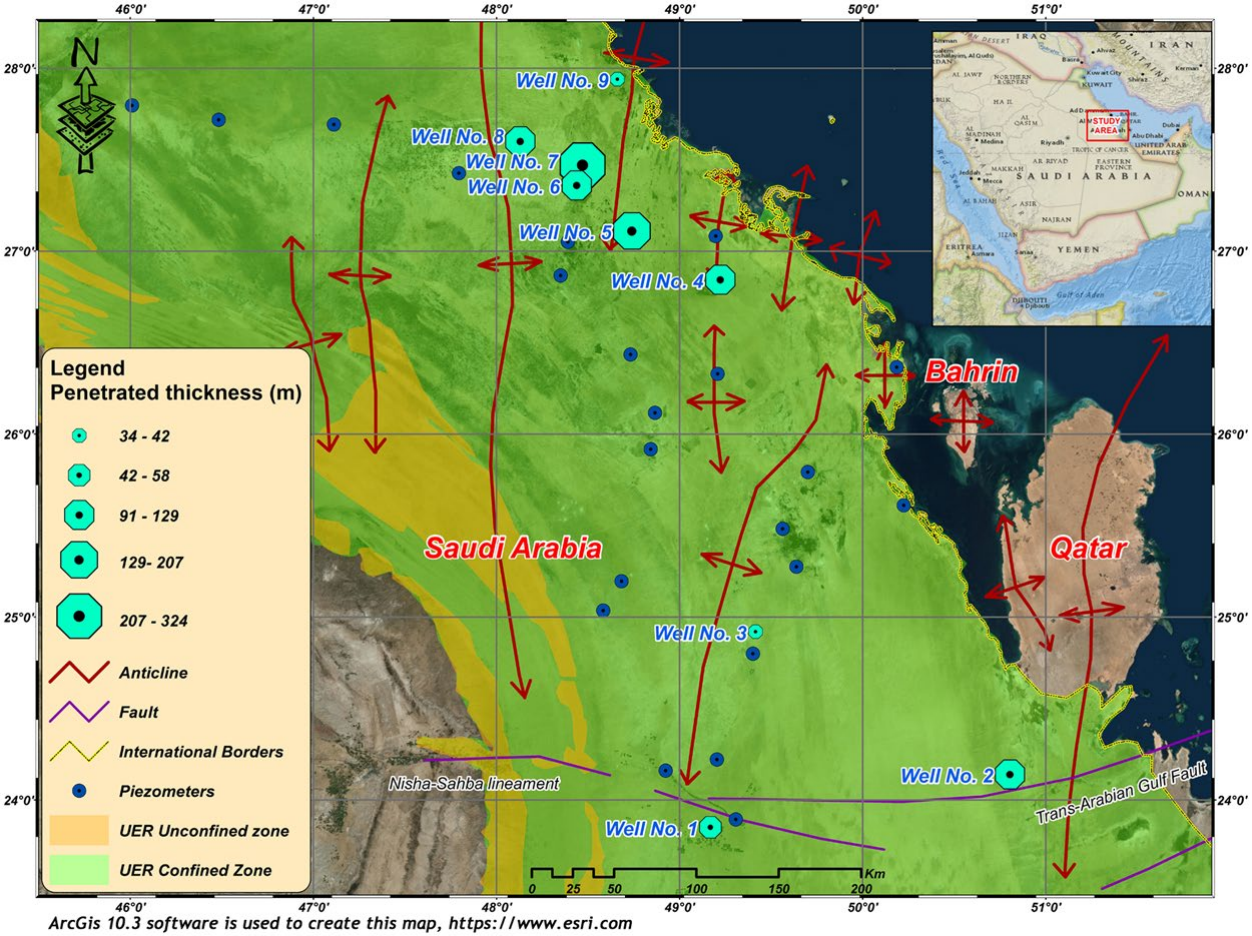
Penetrated thickness of wells tapping the UER aquifer in the study area. The thickness of the UER aquifer increases in the synclinal areas (see location of wells 6 and 7). ArcGIS 10.3 software is used to create this map, http://www.esri.com.

Lower values of K were recorded in the synclinal areas; however, greater thicknesses of carbonate rocks are found at greater depths with low porosity and permeability values (Fig. 12). The estimated T values of the UER aquifer vary between 4.1 × 10^−5^ and 2.8 × 10^−1^ m^2^/s, with an average of 4.2 × 10^−2^ m^2^/s (Fig. 13 and Table 3). Figure 13 shows that the high T values were recorded on or near the anticlinal axes, where the fracture and joint densities are high, while the lower values were recorded in the synclinal troughs. Although the maximum penetrated thicknesses were found in the synclinal areas, the lowest T values were also recorded here. This is because UER aquifer has the lowest K values in these synclinal areas (Figs 13 and 14).

The estimated S varies between 2.1 × 10^−6^ and 8.8 × 10^−4^ with an average of 2.1 × 10^−4^. The wide difference between these values is because of the existence of permeable caverns as a result of the karstification process associated with the development of fractures, particularly above the anticlinal zones, and the expansion of the water which is attributed to the compression of the aquifer. Variations in the carbonate aquifer media result in changes in K and hence T and yield.

## Discussion

**Figure 15:**
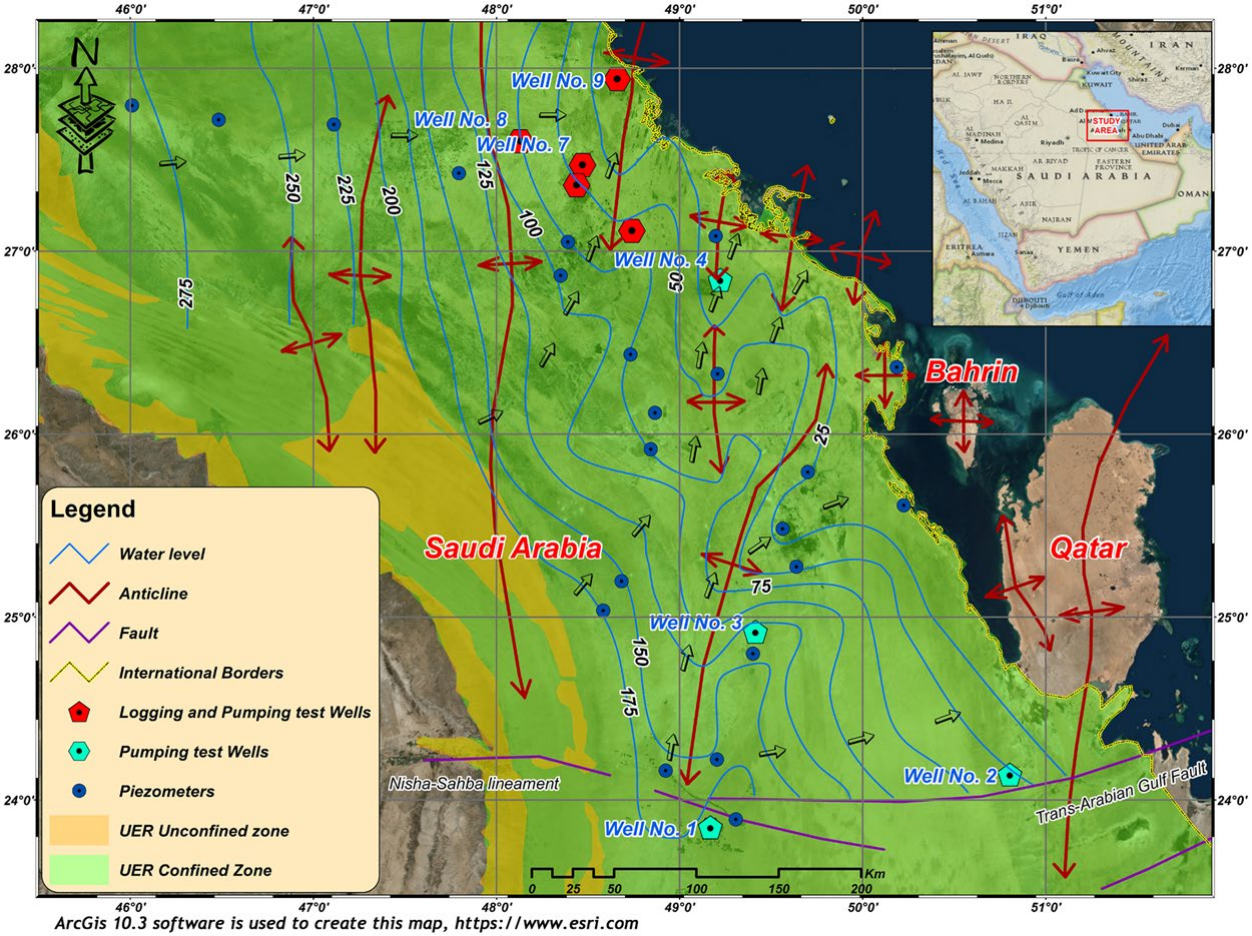
A potentiometric surface map of the UER aquifer in the study area. The directions of groundwater flow are indicated by the small green arrows. The groundwater flow seems to be forced through the axes of the broad anticlinal system. ArcGIS 10.3 software is used to create this map, http://www.esri.com.

In the study area, the groundwater flow system is influenced by a combination of topography, hydrogeological setting, and geological structures (Fig. 15). In the western part at the outcrop areas of the UER aquifer, an unconfined condition prevails. In the east, there is a transitional zone that extends until the piezometric level intersects with the confining Rus, Dammam, and Neogene formations. Far to the east, near the Arabian Gulf, the UER aquifer exhibits a confined condition. Near the Arabian Gulf, these rocks are found at great depths with low porosity and permeability. In these areas, there are inland coastal sabkhas, which are thought to be mainly fed by the upward leakage of the UER aquifer groundwater.

Groundwater of the UER aquifer has a regional flow controlled by the distribution of hydraulic conductivity; however, groundwater flows from areas of recharge (west) to areas of discharge (east). Groundwater levels vary between more than 275 m above sea level near the outcrop areas to sea level at the Arabian Gulf, eastward, along a horizontal distance of 230 km, generating a non-uniform groundwater hydraulic gradient of approximately 0.005 on average. There are some local flow patterns, which are influenced by geological structure (Fig. 15). In the northern area, groundwater flow is slightly influenced by geologic structures, while it is highly influenced by the folding system in the remainder of the study area. The fold system forms chains of asymmetric anticlines and synclines, whose axes plunge to the N and NE. These folds force the groundwater flow system to deviate from a W-E directional flow to a SW-NE meandering flow pattern; however, varying directions in the maximum and medium K follow the strike and dip of bedding, respectively, while the minimum K direction is perpendicular to the bedding^3^. The broad anticlinal system forces groundwater flow through anticline axes and in places it overflows from one to a nearby anticline (Fig. 15). Notably, well No. 5, where a good fracture system is indicated from the SGR geophysical logs (see Fig. 7), is along one of the indicated SW-NE flow lines (see Fig. 15 for well location and flow directions).

Moreover, the groundwater flow in UER aquifer is highly influenced by the large variation in porosity and hydraulic conductivity in the anticlinal structure areas, where karstic features are identified. Smaller salinity and relatively younger groundwater age of UER aquifer are recorded by GTZ (2006) on or near the anticlinal structure areas rather than synclinal areas. Existence of some inland springs (Al-Hofuf and Al-Ahsaa) as well as submarine springs beneath Persian Gulf along the extension of these anticlinal axes is a support of this conclusion. A large part of this natural discharge occurs where there is a direct upward discharge from the UER aquifer into the overlaying Rus, Dammam and Neogene aquifers through the eroded anticline crests and adjacent fractures and faults (Fig. 15). The flow directions show good correlation with the structural lineament map derived from the 3D seismic interpretation carried by Al-Ali and Stenger (2001)^41^.

Published information regarding production wells in the study area has indicated that production screens were installed most commonly in the front of the middle and lower zones of the UER aquifer to attain the maximum water column in order to increase aquifer transmissivity^38^. Correlation with the results obtained from the detailed petrophysical analysis in this study has showed that these aquifer zones have the lowest hydraulic properties and modest petrophysical properties. For any further well drilling/production activity, we strongly recommend placing the screens in the upper and middle zones (Z1 and Z2) of the UER aquifer with a priority to the upper zone. Therefore, the implied hydrogeophysical technique and its associated detailed petrophysical analysis helped in improving our understanding regarding the characteristics of the UER aquifer in this particular area of the eastern part of Saudi Arabia, where many complex geological and hydrogeological aspects occur. The implied approach can help in characterizing similar karstic aquifer systems and improve strategies for identifying the most productive zones.

## Methods

A large number of wells were drilled in the study area to discharge groundwater from the UER aquifer mainly for demotic and agricultural purposes. Nine wells were selected to investigate the hydraulic properties of the UER aquifer and perform the necessary well logging analyses (Fig. 1). In addition, 22 piezometers were used to map the hydrological setting and groundwater flow. Two main approaches were mainly utilized in this study. The first approach included performing a comprehensive well-logging analysis to detect the main petrophysical properties of the UER aquifer. Special emphasis was given to detect the hydraulic parameters of the aquifer. Other important parameters that have a direct influence on the hydraulic properties of the UER aquifer were also determined, such as those of the fracture system and possible lateral flow. The second approach was dependent on analyzing the pumping test data to determine the T, K, and S of the UER aquifer by applying the examined well-fitted models of the leaky and partially penetrated confined aquifers^4,42^.

### Data Scaling

Scaling of the local meter-sized volume analysis given by well logs to large-scale aquifer pump tests was done. Nine (9) wells were tested for pumping test; out of them five (5) were subjected to both pumping test and well log analyses (Wells No. 5, 6, 7, 8 and 9). For data scaling, the log-derived hydraulic properties of the UER aquifer were statistically averaged and compared with those obtained from pumping test analysis. An integrated correlation was conducted between the log-derived and pumping test results to provide a good matching between both categories of results. The estimated hydrogeologic parameters were mapped in relation to the prevailing structural pattern of the area to detect possible underground fluid flow.

### Petrophysical Analysis

The geophysical well logs of the available deep wells within the study area were used mainly in performing a number of qualitative and quantitative well-logging analyses. The analyzed logs include short and long normal resistivity logs, induction logs, neutron, density, computed gamma ray (CGR), spectral gamma ray (SGR) and caliper logs. An academic license of the Interactive Petrophysics software (IP v.4.10) was used in conducting the necessary analyses and plots.

#### Analysis of gamma ray logs

The natural gamma-ray log (GR) can be utilized as a shale volume indicator (Vsh) as well as for detecting the clay type and facture system^43–45^. The first step is to estimate the index gamma ray^46^. Several relationships have been developed and applied for estimating shale volume. (See Supplementary Information for more details)^47–49^.

Both of the spectrometry gamma ray log SGR (thorium ‘Th’, ppm, uranium ‘U’, ppm, and potassium, %)) and the computed gamma ray log CGR (Th, potassium) were used also in the analysis. They have been widely used in evaporites and carbonate reservoirs to highlight its fracture system. The difference between the SGR and CGR curves is usually interpreted as a function of U content, since the first is composed of three elements (Th, potassium, U) and the second is composed of two elements (Th, potassium)^50^. The U content was estimated as follows:

1 $${\rm{U}}\,{\rm{content}}={\rm{SGR}}-{\rm{CGR}}$$

A wider separation between SGR and CGR curve overlays, if associated with high caliper readings, will reflect the U content that might be accumulated in the fracture system. Thus, possible zones of good lateral flow and K can be indicated.

#### Estimation of porosity

Total porosity is volume of the pore spaces to the total volume of the rock, while effective porosity accounts only to the interconnected pore spaces. Neutron and density logs seem to be the most appropriate for use in total porosity estimation supposing that the lithologic and matrix parameters are known^51^. The effective porosity is further generated after correcting for the shale effect^51–53^. The following equations were used for total and effective porosities estimation from neutron log:

2 $$\varnothing {N}_{Tot}=\varnothing N\,log\,$$

3 $$\varnothing {N}_{eff}=\varnothing {N}_{Tot}\ast Vsh$$

where, *ϕN*_*Tot*_ is the total porosity from neutron log (fr), *Vsh* is the shale content from GR (fr), and *ϕN*_*eff*_ is the neutron effective porosity (fr).

#### Fluid content

In the study area, the UER aquifer was found to be fully saturated with water, where confined conditions were prevailing. Although this is the situation, the subject of saturation was discussed because not all the contained water can be produced. Part of the aquifer water (which was supposed to be 100%) cannot be produced (connate water). The Archie equation was used for fluid content determination and differentiation between producible and connate water^54^. It has been applied for clean lithology (no shale) as follows:

4 $$S{w}^{n}=(\frac{a}{{\varnothing }^{m}}.\frac{Rw}{Rt})$$

where, *Sw* is the water saturation, *a* is a constant, *m* is the cementation factor, *ϕ* is the effective porosity (p.u.), *Rw* is the water resistivity, *Rt* is the true aquifer resistivity, and *n* is the saturation exponent.

Pickett plot (1972)^55^ is another simple technique that has been applied for estimating *Sw*, *Rw*, *m* and *n*. It is a graphical solution of Archie equation, in a rearranged form, where *Rt* is plotted against the porosity on a log-log paper. The constant *a* is often equal to unity, while the saturation exponent *n* and cementation factor *m* have a wide range of values in carbonate rocks^51,55,56^.

Since condensable content of shale was recorded at UER aquifer, another saturation model was applied to account for the presence of this conductive shale, where the apparent formation factor and apparent water resistivity (*Rwa*) were used^40,49^. The model solved for water saturation detection without regard to the manner of shale distribution (See the Supplementary Information for more details).

In addition, permeability (k) was generated using Timur formula^57^ based on the porosity and water saturation (irreducible). The storativity (S), hydraulic conductivity (K) and transmissivity (T) were derived from the borehole geophysical data^58–60^. A wide range of K was found for carbonate aquifers and other sedimentary rocks (Table 2). More details about the technical details of these parameters and the used equations were represented in the Supplementary Information provided at the end of the article (Equations S4-S7).

### Pumping Test Analysis

Constant rate and long-duration pumping tests of nine wells were used to determine the T and K of the UER aquifer by applying the examined well-fitted models of both a leaky aquifer and a partially penetrated confined aquifer. Hantush (1961)^4^ derived equations that incorporate the Hantush and Jacob method to include the effects of partial penetration in a leaky confined aquifer. The model of Hantush and Jacob (1955)^42^ represents flow to a fully penetrating line sink discharging at a constant rate in a homogeneous, isotropic, and leaky confined aquifer of infinite extent, described as follows:

5 $$s=\frac{Q}{4\pi T}w(u,\frac{r}{B})$$

where, Q is the pumping rate, T is the transmissivity, and w(u, r/B) is the Hantush and Jacob well function for leaky confined aquifers.

The model of Hantush (1961) is a partially penetrating pumping well that produces the vertical components of the flow in the pumped aquifer. The following equation is applicable:

6 $$\begin{array}{rcl}s & = & \frac{Q}{4\pi T}[w(u,\frac{r}{B})+\,\frac{2{b}^{2}}{{\pi }^{2}\,(1-d)(l\text{'}-d\text{'})}\sum _{n=1}^{\infty }\frac{1}{{n}^{2}}(sin(\frac{n\pi l}{b})-sin(\frac{n\pi d}{b}))\,\\  &  & .\,(sin(\frac{n\pi l\text{'}}{b})-sin(\frac{n\pi d\text{'}}{b})).w(u,\,\sqrt{{(r/B)}^{2}+{K}_{z}/{K}_{r}{(\frac{n\pi r}{b})}^{2}}\,)]\end{array}$$

7 $${\rm{\beta }}=\sqrt{{(r/B)}^{2}+{K}_{z}/{K}_{r}{(\frac{n\pi r}{b})}^{2}}$$

where, *b* is the aquifer thickness, *d* is the depth to the top of the pumping well screen, d′ is the depth to the top of the observation well screen, *K*_*r*_ is the radial (horizontal) hydraulic conductivity, *K*_*z*_ is the vertical hydraulic conductivity, *l* is the depth to the bottom of pumping well screen, *l′* is the depth to the bottom of observation well screen, *w(u*, *β)w(u*, *β*) is the Hantush and Jacob well function for leaky confined aquifers, and *z* is the piezometer depth.

## Conclusions

In this study, available geophysical well logging data and long-duration pumping tests were integrated and processed to obtain the detailed hydrogeological characteristics of the UER aquifer in the eastern coastal area of Saudi Arabia and to demonstrate the influence of geological structure on groundwater flow patterns. Scaling of the local well logs to large-scale aquifer pump tests was done, where nine wells were tested for pumping test and well log analyses (5 wells). The log-derived hydraulic properties of the UER aquifer were statistically averaged and compared with those obtained from pumping test analysis. The following are the main conclusions:

- The UER aquifer was found to be a leaky confined to confined aquifer with permeable zones because of the karstification process associated with the development of fractures, particularly above the anticlinal structures.
- The well logging analyses showed that both limestone and dolomite constitute the main lithological components of the UER aquifer sharing 50–60% of the volumetric analysis of the aquifer, while the remainder (40–50%) is represented by the shale volume and pore space. The overlying Rus Formation is composed mainly of soft evaporites that showed low porosity and K.
- Detailed petrophysical analysis showed that the UER aquifer can be divided into three zones. Two zones (Z1 and Z3) of considerable thickness at the upper and lower sections of the aquifer, and one middle thicker zone (Z2). Good petrophysical and log-derived hydraulic properties were assigned for the upper zone as compared to the two underlying zones.
- A good fracture system signature with possible lateral flow was indicated from the SGR and CGR curve overlays in front of the upper zone of the UER aquifer and the overlying Rus Formation.
- Different scale logs were integrated to determine the hydraulic parameters (T, K, and S) while long-duration pumping tests (local-to-regional measurements) were used to validate the well-logging analyses. The fitted models of tested pumping wells showed that the T, K, and S values of the UER aquifer are moderate and decrease with depth.
- An integrated correlation was conducted between the log-derived and pumping test results to provide a good matching between both categories of results.
- The estimated hydraulic parameter values acquired using the integrated interpretation of the logging and pumping test data were within the expected range for the confined and leaky confined aquifers. The obtained results confirmed the possibility of obtaining a detailed satisfactory estimation of hydraulic parameters using well-log analyses, which was validated using the results of the long-duration pumping test.
- The estimated hydrogeologic parameters were mapped in relation to the prevailing structural pattern of the area to detect possible underground fluid flow. Near the coastal area, groundwater flow is influenced by the fold system, which forces groundwater to deviate from a W-E to a SW-NE direction in a meandering flow pattern. The broad anticlinal system forces groundwater flow through anticlinal axes and in places it overflows from one to a nearby anticline.
- This approach can offer detailed valuable information regarding structural influence on groundwater flow and obtain detailed hydraulic characteristics of the different aquifer zones. It can enhance local existing models of confined and leaky aquifer systems, and improve strategies for identifying the most productive zones, and consequently, reduce well-drilling costs.

## Supplementary information

SREP-18-17576B-Supplementary Information File
