# Supplementary material for: Quantitative hydro-geophysical analysis of a complex structural karst aquifer in Eastern Saudi Arabia
Source: Sci Rep. 2019 Feb 26;9:2825. doi: 10.1038/s41598-019-39192-4 (PMC6391462; doi:10.1038/s41598-019-39192-4)
Supplement: Supplementary file 1 — SREP-18-17576B-Supplementary Information File [file 41598_2019_39192_MOESM1_ESM.docx]

**Quantitative hydro-geophysical analysis of a complex structural karst aquifer in Eastern Saudi Arabia**

Mohamed El Alfy^1,3^, Aref Lashin^2, 4*^, Turki Faraj^1^, Abed Alataway^1^, Qassem Tarawneh^1^, Abdelaziz Al-Bassam^5^

^1^Prince Sultan Institute for Environmental, Water and Desert Research, King Saud University, Riyadh 11451, Saudi Arabia

^2^Petroleum and Natural Gas Engineering Department, College of Engineering, King Saud University, Riyadh 11421, Saudi Arabia

^3^GeologyDepartment, Faculty of Science, Mansoura University, Mansoura, 35516 , Egypt

^4^Geology Department, Faculty of Science, Benha University, Benha, 13518, Egypt

^5^ National Center for Water Research and Studies (NCWRS), MEWA, Saudi Arabia.

**^*^***Corresponding author (arlashin@ksu.edu.sa)*

**Shale volume estimation**

The following is the most common equation that was used from shale volume estimation is:

$$Vsh = \frac{GR-GR min}{GR max- GR min} (S1)$$

*Vsh* is the shale volume (fr), *GRmin*is the minimum GR reading,* GRmax* is the maximum GR in front of shale.

**Fluid content shaly zones**

A modified form of Archie equation has been applied to accommodate for the presence of shale, which act as another electrically conductive material beside the formation water. The model of the Fertl and Hammack (1971) [40] was applied as follows:

$$Sw= \left( \frac{F*Rw}{Rt} \right)^{0.5}- \frac{Vsh*Rw}{0.4* \emptyset* Rtsh} (S2)$$

where, *F* is actual formation factor, *Rw* is the water resistivity, *Rt* is the true aquifer resistivity, *Vsh* is the shale volume (fr), *Rtsh *is the resistivity of conductive shale.

The apparent water resistivity (*Rwa*) which is a simple rearrangement of the classic Archie equation was used with other logging parameters (*GR*) for aquifer zonation. It is equal to *Rw* in front of the 100% water-bearing zones. It can be identified as follows:

*Rwa = Rt/F* (S3)

[**Storativity**](http://www.aqtesolv.com/aquifer-tests/glossary-of-aquifer-testing-terms.htm#Storativity) **(S)**

For confined aquifers, the value of S varies from 5×10^-5^ to 5×10^-3^ with specific storage and aquifer thickness [58]. For fissured carbonate rock, the compressibility varies from 10^-10^ to 10^-8^ [5]. The S was calculated as follows:

$S=S_{s} .h (S4)$

where, *S* is the [storativity](http://www.aqtesolv.com/aquifer-tests/glossary-of-aquifer-testing-terms.htm#Storativity) (dimensionless), *S_s_*is the specific storage (L^-1^) and *h* is the aquifer thickness (m). Specific storage is related to the compressibilities of the aquifer and water as follows:

$S_{s}=\rho g\left( \alpha+ {\emptyset N}_{eff} . \beta\right) (S5)$

where, *ρ* is the density of the water, *g* is the gravitational acceleration, α is the aquifer compressibility (T^2^L/M), *φN_eff_*  is the effective porosity (fr), and β is the compressibility of water.

**Hydraulic conductivity (K) and Transmissivity (T)**

In the current research, two procedures were applied for K estimation; the first was based on the Kozeny-Carman-Bear equation [6,59,60]. The average K can be calculated as follows:

$K=\left( \frac{g}{\mu} \right)\left[ \frac{d^{2} \emptyset^{3}}{180 (1-\emptyset)^{2}} \right] (S6)$

where, *ρ* is the fluid density, *g* is the gravity acceleration in m/sec^2^; *μ* is the dynamic viscosity, *d* is the grain size, and *φ* is the aquifer porosity.

The K can be also derived using the following equation:

K= k/μf (ρwg/gc) (S7)

where, *k* is the permeability, *ρw* is the density of the formation water, *g* is the gravity acceleration, *gc* is a term equal to 1*.*0, and *µf* is the viscosity of the formation fluid.

The input values for porosity and permeability were based on well-log analyses. Because the top of the UER aquifer occurs at relatively shallow depths, the dependence of K on temperature can be neglected. Transmissivity (T) of the aquifer was further estimated based on the aquifer K and thickness.

**Figure S1.** The petrophysical properties of the UER aquifer as inferred from logging analyses of well No. 6. No pore system or fluid content are indicated in front of the salt section of the Rus Formation.

**Figure S2.** The hydraulic properties of the UER aquifer as inferred from logging analyses of well No. 6. Tracks 5 and 6 represent the log-derived K, S, and T, respectively (see Table 3 for associated values). The aquifer bulk volume of water is indicated in Track 7.
